# Surgical outcome of collateral ligament injury with metacarpal head fracture in a near amputation after power saw injury: Case report

**DOI:** 10.1016/j.ijscr.2019.10.078

**Published:** 2019-11-02

**Authors:** Wongthawat Liawrungrueang

**Affiliations:** Department of Orthopedics, Faculty of Medicine, Prince of Songkla University, Thailand

**Keywords:** Power saw injury, Collateral ligament injury, Near amputation

## Abstract

**Introduction:**

The incidence of thumb amputation is high in developing and industrialized countries. A power saw injury is very traumatic to the soft tissue and neurovascular system, and thus difficult for orthopedic and plastic surgeons to treat.

**Presentation of case:**

A 41 year old male injured his right hand with a power saw. The poor condition of the soft tissue and collateral ligament damage to the head of the metacarpal fracture meant that near amputation was a possibility. The patient received adequate antibiotic and was taken to the operating room for fixation with convergent screws system, repaired and reconstructed collateral ligament complex. At 3 month follow up, author saw the union of the intraarticular fracture and the metacarpophalangeal joint (MCP). The patient was followed up at 1 year, the sensory and function was full recovered. The patient was extremely satisfied with this treatment and can now work normally again.

**Discussion:**

The collateral ligament complex injury with metacarpal head fracture in a near amputation after power saw injury; it is a very unstable injury. In this case, author performed an early adequate intravenous antibiotics in the emergency room and was taken to the operating room for repair and reconstruction. Neurological status and hand function were recovered that patient was extremely satisfied in this surgical planning.

**Conclusion:**

The power saw injury is a severe injury which requires special attention for treatment. Furthermore, the early reparation, reconstruction and fixation with screws in a convergent system is the treatment of choice in this condition.

## Highlights

• Case report of collateral ligament injury with metacarpal head fracture in a near amputation after power saw injury.
• Special attention for treatment. The early reparation, reconstruction and fixation with screws in a convergent system procedure was the treatment of choice in this condition.
• Intra-articulation union of a metacarpal head fracture and the good function of metacarpophalangeal joints (MCP) joint at the 1-year follow-up.

## 1 Introduction

Nowadays, power saw injury is the most common injury in steelworkers and woodworkers. In the past, power saw injuries accounted for 6% with a reported incidence of 2.6 per 1000 person-hours [1,2]. The standard treatment is repair, reconstruction, replantation and revascularization in near amputation or amputation cases. However, in many cases with severe injury the patients choose a closed stump in order to return to work quickly. The author presents a treatment of a traumatic and near amputation of the index finger with collateral ligament injury with metacarpal head fracture. The work has been reported in line with the SCARE criteria [3].

## 2 Presentation of case

**Fig. 1:**
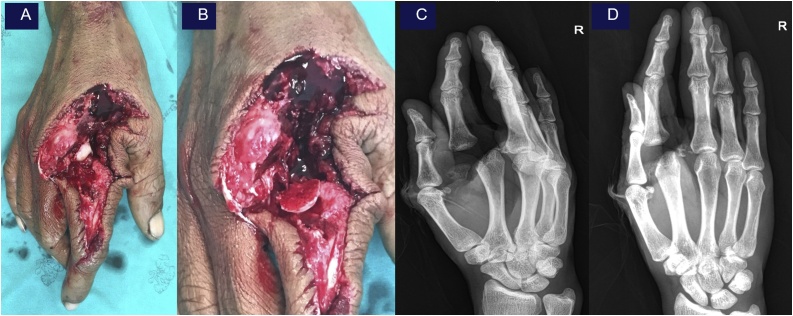
Pre – operative examination showing nearly amputation of right index (Fig. 1A). Radial of digital artery, vein and nerve of index completely tear with the articular surface of metacarpal head fracture (Fig. 1B). The radiographs show fracture dislocation of the head of the second metacarpal with articular involvement fracture 25% in oblique view (Fig. 1C) and AP view (Fig. 1D).

A 41-year-old left-hand dominant male general worker injured his right hand with a power saw while working. He presented to the emergency department within 15 min after injury, along with the nearly amputated of his index finger. The emergency doctor team prepare for preoperative lab and consult orthopedic team for revaluation and proper management. Examination of his right hand found a dirty shearing cut twelve-centimeter wound on the radial side of the index finger extending to first web space to a point just distal to the metacarpophalangeal joint (MCP) joint, with nearly amputation (Fig. 1A). The nearly amputated segment contained with MCP radial collateral ligament, extensor tendon injury (zone V), radial of digital artery, vein and nerve of index completely tear with the articular surface of metacarpal head fracture (Fig. 1B). The patient’s neurovascular examination was decreased in sensation and numbness on the radial side of the distal index finger by the pinprick test. Motor examination showed the loss of some part of extensor muscle and no soft tissue coverage. Capillary refill was about 2 s (impaired). The radiographs show fracture dislocation of the head of the second metacarpal with articular involvement fracture 25% (Fig. 1C–D). In the Emergency Department, tetanus toxoid was booster, cefazolin and gentamicin were administered, and the patient was taken to the emergency operating room.

The operative findings show open fracture dislocation of the 2^nd^ MCP joint of right hand with metacarpal head fracture and open fracture base of proximal of the 2^nd^ finger at ulnar lip with intra articular involvement 25%, capsule of the 2^nd^ MCP joint tear, completely tear of extensor digitorum communis (EDC), radial and ulnar both collateral ligament tear, completely tear digital nerve of the 2^nd^ finger at ulnar side, tear of the 1^st^ and the 2^nd^ interosseous muscles.

**Fig. 2:**
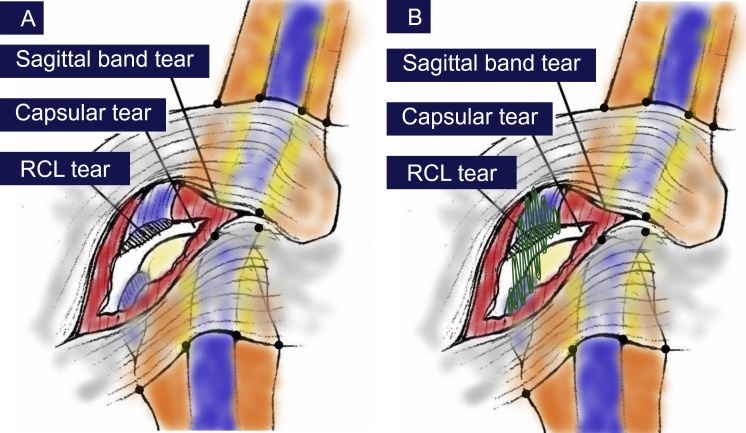
Intra-operative collateral ligament complex injury (Fig. 2A) and continuous intraosseous suture which imbedded in the radial side of the metacarpal head (Fig. 2B).

The operative procedure was performed. The fragment of metacarpal head fracture was reduced and fixed with 1.3 mm 2 cortical screws in convergent system and then checked under fluoroscope. The collateral ligament was identified as illustration (Fig. 2A). The tear of the radial sagittal bands, the conjoined origin of the radial collateral and accessory collateral ligaments was seen at surgery. The joint alignment was restored by repair of the conjoined origin of the collateral and accessory collateral ligaments to a continuous intraosseous suture which imbedded in the radial side of the metacarpal head with non-absorbable suture (Ethibond 4-0) (Fig. 2B). Capsular repair was done with non-absorbable suture.

**Fig. 3:**
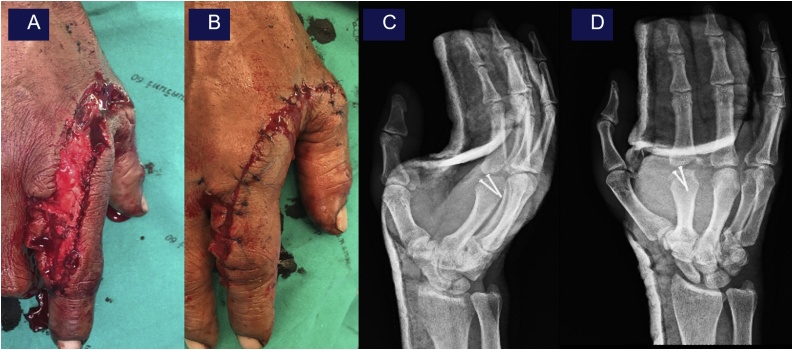
Intra-operative repair collateral ligament complex (Fig. 3A-B) and post – operative showing post-operative x-ray oblique view (Fig. 3C) and AP view (Fig. 3D).

Ulnar lip of base of the 2^nd^ finger was reduced. EDC was repaired with 4-strand-modified Kessler technique with non-absorbable suture with epitendinous suture. The digital artery was identified and intact at the ulnar side but unable to repair at the radial side due to large gap and segmental injury. Digital nerve at the ulnar side was identified for proximal and distal part and was end to end repaired. The 1^st^ and the 2^nd^ interosseous muscles were repaired with absorbable suture. Skin margin was debrided and the wound was sutured (Fig. 3A-B). Finally, alignment and fixation were checked of all constructs were done under fluoroscope then volar slab was applied in safe position and post-operative x-ray was checked again (Fig. 3C-D).

**Fig. 4:**
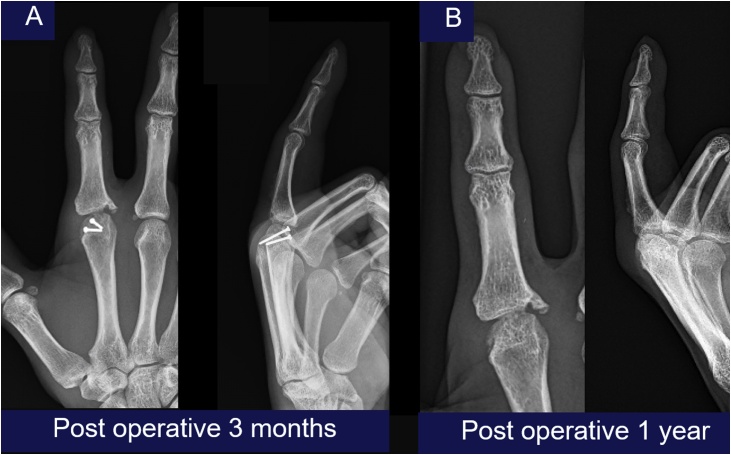
The radiographs showed the union of intraarticular fracture at 3 month follow up (Fig. 4A) and 1 year follow up (Fig. 4B).

**Fig. 5:**
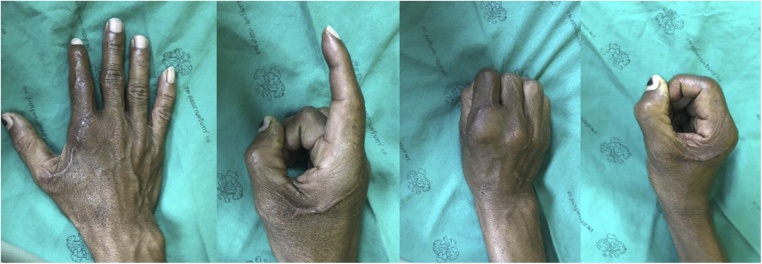
Post – operative examination at 1 years showing sensory was recovered and he had the full flexion and extension of MCP but he had minimal, limited extension of PIP and DIP.

Postoperatively, intravenous cefazolin and gentamicin was continued for 72 h, and the patient was discharged home. The MCP was immobilized in a splint in safe position for two weeks, followed by a course of physical therapy for the range of motion. The patient returned to light duty work at six weeks with continued hand rehabilitation protocol. When he returned to follow up 3 months later show union of intraarticular at fracture site (Fig. 4A). He was taken to the minor operating room for removing screws and passive manipulation under local anesthesia and continuous post rehabilitation protocol for preventing stiffness of MCP. Followed up one year later, the x-ray was showed the union of intraarticular fracture (Fig. 4B) and the sensory was recovered and had the full flexion and extension of MCP but he had minimal, limited extension of proximal interphalangeal joints (PIP) and distal interphalangeal joints (DIP) (Fig. 5). The patient was highly satisfied with this treatment and can now work normally again.

## 3 Discussion

The knowledge of the anatomical characteristic of the metacarpophalangeal joint and collateral ligaments is very important. Gaston et al. [4] reported on 14 cases of collateral ligament injuries. All his patients were more than 36 years of age. All those who failed conservative treatment were more than 30 years old. In this group the radial collateral ligament was more involved. The type of conservative treatment did not differ between those who failed conservative treatment and those who did not. The complete ruptures of the MCP collateral ligaments of the finger is more frequent in worker and emergency to surgery treatment [5].

D.J. Shewring and R.H. Thomas [6] reported 19 patients with collateral ligament avulsion fractures from the metacarpal heads of the fingers were treated. Reported eight patients were conservative treatment and eleven patients with displaced fractures were treated by primary internal fixation using a single lag screw through a dorsal approach. Seven of these achieved a full range of movement of the injured digit by 3 months. Four patients failed to regain full flexion of the metacarpophalangeal joint. One patient with a displaced and comminuted fracture was treated with internal fixation at 8 weeks.

In this case, the collateral ligament and the intra-articulation of the metacarpal head fracture were injured by a power saw. The numerous clinical symptoms and various organs involved were difficult to manage with all the structural injuries. Initial intravenous antibiotics and tetanus toxoid were administered, following management guidelines [7].

## 4 Conclusion

Collateral ligament and the intra-articulation of the metacarpal head fracture were injured by a power saw. It was a severe injury which requires special attention for treatment. Furthermore, the early reparation, reconstruction and fixation with screws in a convergent system procedure is the treatment of choice in this condition. This was a case of near amputation after surgical treatment with convergent screws that shows intra-articulation union of a metacarpal head fracture and good function at the 1-year follow up.

## Sources of funding

This research did not receive any specific grant from funding agencies in the public, commercial, or not-for-profit sectors.

## Ethical approval

The patient provided written informed consent to have the case details and any accompanying images published. Prince of Songkla University Institutional Review Board, Faculty of Medicine, Songklanagarind Hospital, Prince of Songkla University (IRB number REC: 62-228-11-4) provided its approval to publish the case details. The patient had the opportunity to refuse. The patient’s personal information remains conﬁdential. There was no cost or harm to the patient as a result of the study.

## Consent

Written informed consent was obtained from the patient for publication of this case report and accompanying images. A copy of the written consent is available for review by the Editor-in-Chief of this journal on request.

## Author contribution

Dr. Wongthawat Liawrungrueang contributed in medical record review and literature search and writing of the draft.

## Registration of research studies

Researchregistry5108.

## Guarantor

Author has read and approve the manuscript and accept full responsibility for the work.

## Provenance and peer review

Not commissioned, externally peer-reviewed.

## Declaration of Competing Interest

Author has no conflict of interest to disclose.
